# Freeze-Dried Camelina Lipid Droplets Loaded with Human Basic Fibroblast Growth Factor-2 Formulation for Transdermal Delivery: Breaking through the Cuticle Barrier to Accelerate Deep Second-Degree Burn Healing

**DOI:** 10.3390/ph16101492

**Published:** 2023-10-20

**Authors:** Hongtao Gao, Xue Wang, Hao Wu, Yuan Zhang, Wenxiao Zhang, Zuobin Wang, Xin Liu, Xiaokun Li, Haiyan Li

**Affiliations:** 1 Hainan Yazhou Bay Seed Laboratory, Sanya Nanfan Research Institute of Hainan University, Sanya 572025, China; 2 College of Tropical Crops, Hainan University, Haikou 570288, China; 3 Engineering Research Center of the Chinese Ministry of Education for Bioreactor and Pharmaceutical Development, College of Life Science, Jilin Agricultural University, Changchun 130118, China; 4 International Research Centre for Nano Handling and Manufacturing of China, Changchun University of Science and Technology, Changchun 130022, Chinawenwen200452@613.com (W.Z.);; 5 School of Pharmaceutical Sciences, Wenzhou Medical University, Wenzhou 325035, China

**Keywords:** CLD-hFGF2, percutaneous delivery, intercellular lipid layer, cuticular barrier

## Abstract

Transdermal administration of chemo therapeutics into burn healing may be an effective treatment to reduce toxic side effects and improve patient compliance for burns. As a transdermal delivery system, Camelina lipid droplets (CLDs) have received great attention due to their biocompatibility, high drug payload, and rapid absorption. However, the absorbed-related mechanisms of Camelina lipid droplets have not yet been reported. Thus, this paper not only demonstrated that CLD can accelerate skin burn healing through promoting hFGF2 absorption, but also elucidated the mechanism between the skin tissue and keratinocytes using Franz, HE staining, DSC, FTIR spectroscopy, and atomic force microscopy with the presence of CLD-hFGF2 freeze-dried powder. We found that the cumulative release rate of CLD-hFGF2 freeze-dried powder was significantly higher than that of free hFGF2 freeze-dried powder into the skin. At the same time, CLD can change the structure and content of lipids and keratin to increase the permeability of hFGF2 freeze-dried powder in skin tissue. Unlike the free state of hFGF2, the biophysical properties of single cells, including height and adhesion force, were changed under CLD-hFGF2 freeze-dried powder treatment. Meanwhile, CLD-hFGF2 freeze-dried powder was more easily taken up through keratinocytes without damaging cell integrity, which provided a new viewpoint for understanding the absorption mechanism with the CLD system for cellular physiology characteristics. Overall, our findings demonstrated that CLD could break through the stratum corneum (SC) barrier and elucidated the transport mechanism of lipid droplets in skin tissue, which provides a crucial guideline in drug delivery applications for future engineering.

## 1. Introduction

A burn is described as a skin injury brought on by intense heat or caustic substances. Burn wounds are a widespread medical issue globally and a cause for 300,000 deaths annually. Burn wounds frequently experience severe bacterial infections and provide an ideal location for the proliferation of bacteria due to the formation of moist necrotic tissue [1]. It affects patients’ health as it prolongs the inflammatory response and hinders re-epithelialization, which delays wound healing as a consequence [2]. Therefore, the question of how to accelerate burn healing is the urgent problem to be solved at present.

Compared with oral administration, transdermal delivery has the ability of long-term drug release and improving patient compliance. Furthermore, the transdermal delivery system was found to be the most acceptable modality of administration in long-term treatment, characterized with a high patient compliance and avoids liver first-pass effects [3]. However, the main barrier preventing the penetration of transdermal drugs is the stratum corneum (SC). To deliver adequate amounts of drugs into the deep skin, various attempts have been made, such as chemical enhancement, osmotic enhancers, ion electroosmotic therapy, microneedle pretreatment, ultrasound, and electroporation [4,5,6,7,8,9]. However, there are several uncertainties regarding the potential toxicity and treatment feasibility of these methods.

With the continuous research and development of techniques, liposomes (lipid droplets) have been approved by the FDA for second-generation transdermal delivery. The plant lipid droplet system was introduced as a new and safe biomaterial that can promote the absorption of drugs through topical and transdermal drug delivery [10,11]. Plant lipid droplets (known as oil bodies) contain a large amount of triacylglycerol inside and are wrapped with a monolayer of phospholipid molecular layers outside, which exhibit a protective effect to maintain the stability of the lipid droplets [12]. Moreover, on the surface of the lipid droplets, the oleosin protein is anchored in a single phospholipid molecule. The two variable amphipathic C- and N-terminal domains of oleosin cover the surface of the lipid droplets. This configuration generates resistance and electrostatic repulsion, keeping lipid droplets as small single units and preventing their coalescence [13,14].

Previous studies have suggested that oleic acid is an effective FDA-approved chemical osmosis accelerator and has been widely used in commercial formulations [15]. Electron microscopic studies have shown that the lipid domain in the cuticle of the skin was stimulated after oleic acid was applied to the skin tissue [16]. The formation of such pools provides permeability defects within the lipid bilayers and thus facilitates the permeation of macromolecules into the deeper epidermal and dermal layers. Coincidentally, Camelina lipid droplets contain many unsaturated fatty acids, such as oleic acid and palmitic acid, which also provide a great convenience for the absorption of foreign proteins in organisms. In addition, as a carrier of transdermal delivery, plant lipid droplets not only have the ability to promote protein absorption, but also have the beneficial characteristics of simple preparation, low cost, few side effects, and easy application.

Camelina [*Camelina sativa* (L.) Crantz] is an oil crop that has been cultivated for 6000 years, and renewed interest was inspired, including that it grows well under a range of agroecosystems and environments [17,18,19]. Its economic value is emerging as an industrial oil raw material, a heart-healthy edible oil, and as an FDA-approved ingredient in animal feed [20,21,22]. In addition, human basic fibroblast growth factor-2 (hFGF2) is an important polypeptide growth factor in organisms. As a national first-class new drug, it is an innovative drug for the treatment of wound repair and chronic ulcer diseases [23,24]. However, as the hFGF2 protein is a highly hydrophilic substance, its absorption was limited, and the effective concentration was greatly reduced in the skin [25].

**Figure 1:**
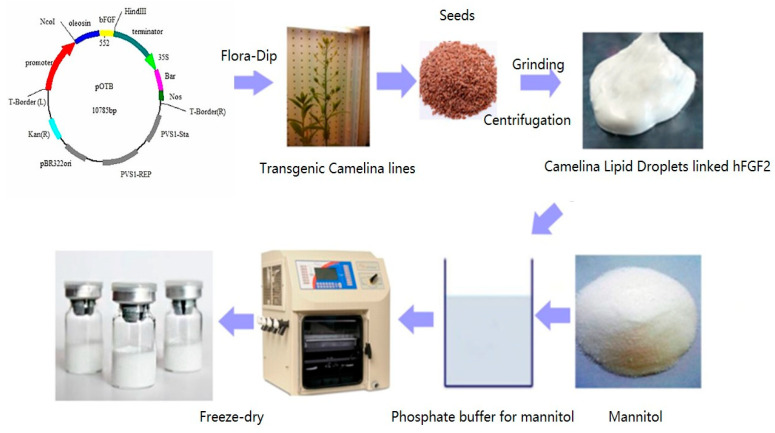
The diagram of freeze-dried Camelina lipid droplets loaded with the hFGF2 formulation.

Therefore, using oleosin fusion technology, hFGF2 was successfully expressed on the surface of Camelina lipid droplets (CLD) in the seeds, and this project developed a natural and long-term storage freeze-dried powder formulation (Figure 1), which can promote the absorption of hFGF2 and prevent the onset of inflammation for accelerating wound healing. In this paper, we found that the cumulative release rate of CLD-hFGF2 freeze-dried powder was significantly higher than that of free hFGF2 freeze-dried powder into the skin. At the same time, CLD-hFGF2 freeze-dried powder was able to change the structure and content of lipids and keratin to increase the permeability of hFGF2 freeze-dried powder and improve the deep second-degree burn wound healing process in the skin tissue. This paper revealed the mechanism of the interaction between CLD-hFGF2 freeze-dried powder and skin epidermal tissue, which helped to obtain valuable information to enhance skin permeability and explore the absorption characteristics of CLD-hFGF2 particles in epidermal tissue. Overall, CLD-hFGF2 freeze-dried powder provides an excellent candidate for the development of new burn-healing agents with a wide feature for the pharmaco-therapeutic application prospect.

## 2. Results

### 2.1. Characterization of CLD-hFGF2 Freeze-Dried Powder

**Figure 2:**
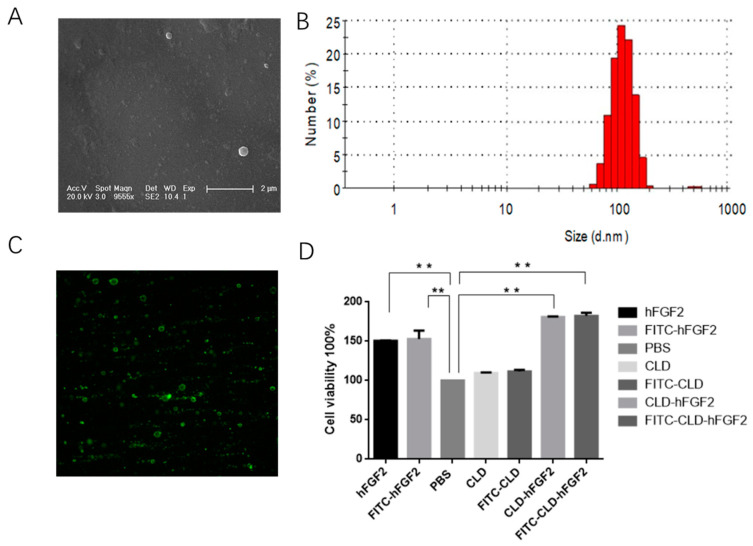
Microstructural observation and activity analysis of CLD-hFGF2. (**A**) Ultrastructural observation of CLD-hFGF2 using TEM; (**B**) analysis of particle size of CLD-hFGF2 with DSL; (**C**) detection of the protein for CLD-hFGF2 freeze-dried powder with Western blot; and (**D**) assay of cell viability of CLD-hFGF2 and CLD-hFGF2 freeze-dried powder. (** *p* < 0.01).

As a safe, natural, and effective transdermal delivery system, the CLD system allows pharmaceutical proteins to be fixed on the C-terminal end of the oleosin protein and be anchored onto the surface of the lipid droplets [26,27]. It enables a simple and convenient separation from the Camelina seeds with a gentle liquid-liquid phase centrifugation process [28,29]. In this study, we successfully prepared an economic and efficient solution of Camelina lipid droplets linked to hFGF2 freeze-dried powder, which has been stably stored at room temperature and breaks the limitation for commercial hFGF2 freeze-dried powder only stored under low temperatures. The nanostructure of Camelina lipid droplets linked to hFGF2 was analyzed using transmission electron microscopy. As shown in Figure 2A,B, the results demonstrated that CLD-hFGF2 freeze-dried powder was uniformly dispersed in a spherical structure, and the particle size was approximately 100 nm, which is beneficial for absorption in the skin tissue. The band of CLD-hFGF2 freeze-dried powder was detected at 34 kD, but the CLD freeze-dried powder did not show the hybridization signal clearly (Figure 2C). Furthermore, the CLD-hFGF2 freeze-dried powder was able to significantly promote cell proliferation more than hFGF2 (Figure 2D).

### 2.2. In Vitro Percutaneous Permeation for CLD-hFGF2 Freeze-Dried Powder

**Figure 3:**
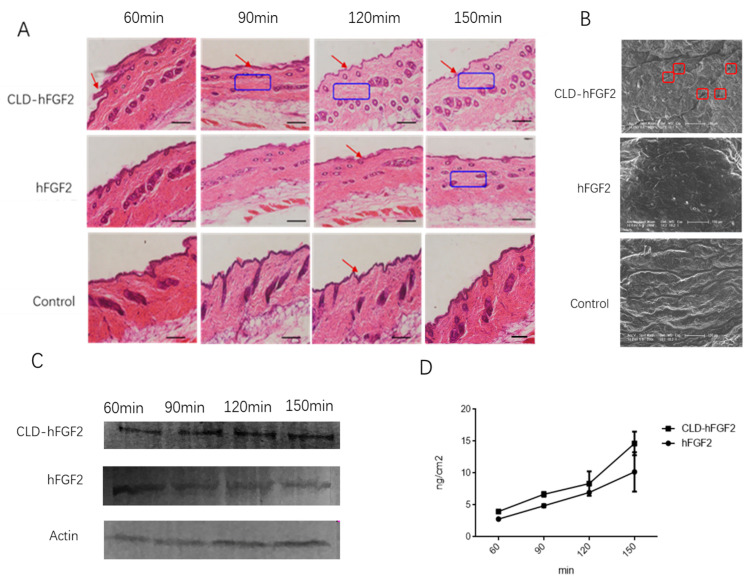
Evaluation of the cumulative release of CLD-hFGF2 in the skin tissue. (**A**) Histological diagram analysis of the skin under the treatment of PBS, CLD-hFGF2, and hFGF2. The red arrow shows the changes of the stratum corneum of the skin tissue for different treatment, the blue boxes show how loose the subcutaneous tissue after different treatment contact with the skin tissue. (**B**) effect of CLD on the stratum corneum of the skin was observed using SEM. The red boxes shows pores in the skin tissue after CLD-hFGF2 was applied to the skin. (**C**) effect of the absorption of CLD-hFGF2 and hFGF2 freeze-dried powder in the skin at different times using Western blots; and (**D**) the cumulative release of hFGF2 and CLD-hFGF2 freeze-dry powder in the skin tissue was measured using ELISA. Data are presented as mean ± SD (*n* = 5; scale bar = 1 µm).

To evaluate the absorption effect of CLD-hFGF2 freeze-dried powder in the skin tissue, the method of HE stains, Western blots, SEM, and Franz diffusion cells were applied. As shown in Figure 3, the structure of the skin stratum corneum was relatively intact with PBS treatment, and the skin tissue became thin and loose with hFGF2 treatment; however, after CLD-hFGF2 treatment, the skin stratum corneum was significantly thinner, the subcutaneous cell space was significantly larger, and the subcutaneous epidermal tissue became loose (Figure 3A). Meanwhile, SEM was used to further observe the changes in the skin tissue, as shown in Figure 3B, where the skin surface structure became loose and porous with the presence of CLD-hFGF2 freeze-dried powder, while hFGF2 freeze-dried powder did not cause similar effects. In addition, after treatment with CLD-hFGF2 freeze-dried powder, the content of hFGF2 in the skin tissue was significantly higher than hFGF2 freeze-dried powder from *E. coli* (Figure 3C). Furthermore, Franz diffusion cells was used to analyze whether CLD-hFGF2 freeze-dried powder could penetrate the stratum corneum to reach the dermis of the skin. According to the results in Figure 3D, with an extension of time, the cumulative release amount under the subcutaneous skin of the CLD-hFGF2 freeze-dried powder group was significantly higher than that observed with the hFGF2 freeze-dried powder group at 150 min, and the cumulative amounts in the CLD-hFGF2 and hFGF2 groups were 18.55 ± 9.64 μg/cm and 9.58 ± 5.72 μg/cm^2^, respectively (*p* < 0.05). Together, these results indicate that CLD not only improve the stability of the hFGF2 protein in the skin tissue to prevent it from being hydrolyzed by trypsin, but also affect skin tissue permeability, thereby promoting the rapid absorption of the hFGF2 protein and making it more conducive to skin tissue wound healing [10].

### 2.3. CLD-hFGF2 Freeze-Dried Powder Changes the Structure of the Stratum Corneum

**Figure 4:**
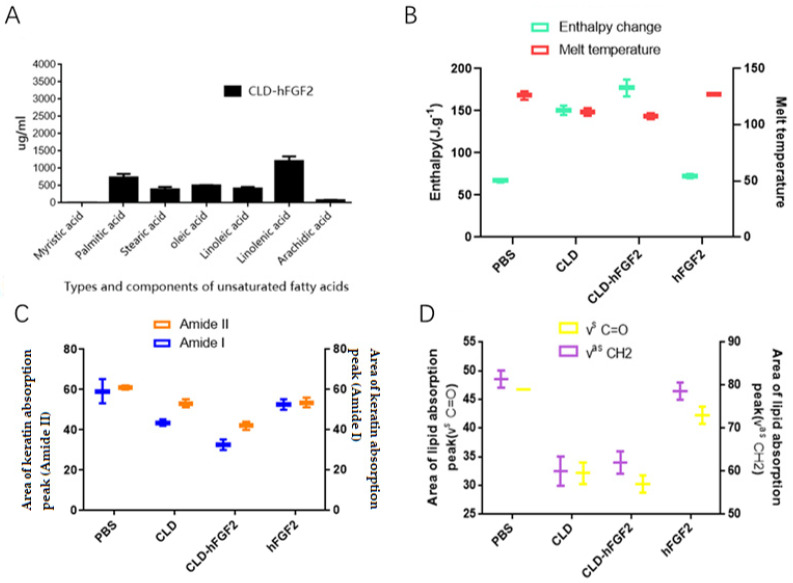
Observation of the skin structure with the treatment of PBS, CLD, CLD-hFGF2, and hFGF2 freeze-dried powder. (**A**) Analysis for the components of the CLD-hFGF2 freeze-dried powder. (**B**) Analysis for the DSC thermograms of skin treated with PBS, hFGF2, CLD, and CLD-hFGF2 freeze-dried powder. (**C**,**D**) Analysis for the FITR of skin treated with PBS, hFGF2, CLD, and CLD-hFGF2 freeze-dried powder.

Several studies have shown that unsaturated fatty acids, such as oleic acid and palmitic acid, have been approved by the FDA as promoters of drug epidermal absorption and have been widely used in commercial formulations [30,31]. To clarify the components of the CLD-hFGF2 freeze-dried powder, the method of HPLC was applied. As shown in Figure 4, the proportions of linolenic acid, oleic acid, palmitic acid and linoleic acid were 26.7%, 15.88%, 13.37%, and 9.51%, respectively. From this finding, it was clear that CLD freeze-dried powder was a natural drug delivery system for accelerating the absorption of hFGF2 in the skin.

The cuticle is the main barrier to drug absorption through the skin. Therefore, the effect of CLD-hFGF2 freeze-dried powder treatment on the cuticle was investigated during the process of absorption. As shown in Figure 4B, compared with the hFGF2 freeze-dried powder group, the change in the melting point and enthalpy for the keratin proteins of the skin was more obvious in the CLD-hFGF2 freeze-dried powder group; these results showed that CLD could change the structure and content of keratin. Meanwhile, the lipid absorption peak (ν^as^ CH2, ν^s^ CH2, and ν^s^ C=O) and keratin absorption peak (Amide I and Amide II) in the skin was further accessed using FITR [32,33,34]. As shown in Figure 3C,D, compared with the PBS group, the characteristic absorption peak of keratin did not change after hFGF2 freeze-dried powder treatment, while the absorption peak of keratin and lipid in the CLD-hFGF2 freeze-dried powder treatment group were significantly shifted, indicating that CLD could change the structure and content of lipids in the skin. In this paper, the characteristic peak areas of keratin and lipid were reduced to varying degrees, especially in the CLD-hFGF2 > CLD > hFGF2 > PBS freeze-dried powder groups. Our experiment results indicated that CLD could cause lipid and keratin structure changes in the skin tissue, which provides a favorable condition for the rapid absorption of the hFGF2 protein in the skin tissue.

### 2.4. CLD-hFGF2 Freeze-Dried Powder Changes HaCat Cell Morphology

**Figure 5:**
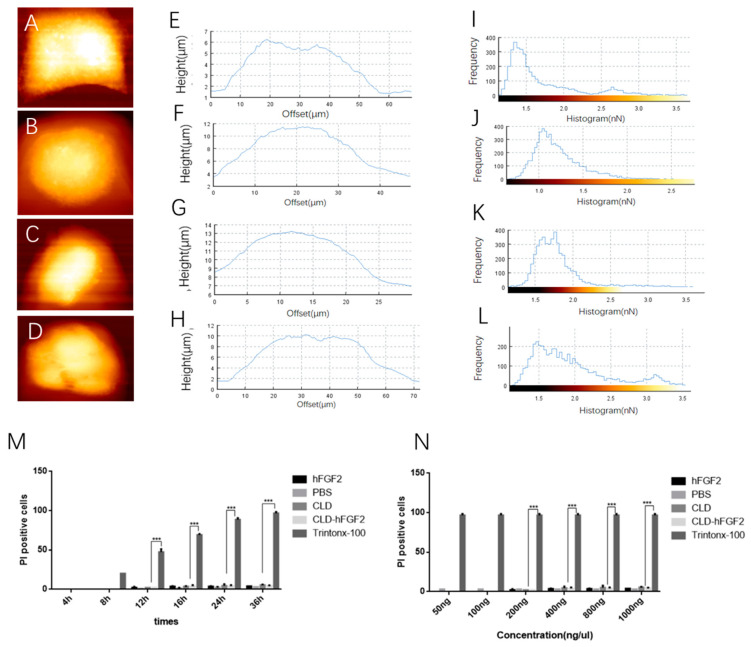
Analysis of HaCat cell morphology after treatment with PBS, CLD, CLD-hFGF2 (100 ng/mL), and hFGF2 (100 ng/mL) freeze-dried powder. (**A**–**D**) AFM images of the HaCat cell height following treatment with PBS, CLD, CLD-hFGF2, and hFGF2 freeze-dried powder; (**E**–**H**) statistical analysis for HaCat cell heights were treated with PBS, CLD, CLD-hFGF2, and hFGF2 freeze-dried powder; (**I**–**L**) statistical analysis of the HaCat cell adhesion force under the treatment of PBS, CLD, CLD-hFGF2, and hFGF2 freeze-dried powder; and (**M**,**N**) evaluation of keratinocyte membrane integrity with PBS, CLD, CLD-hFGF2, and hFGF2 freeze-dried powder treatment at different times and concentrations; Data are presented as the mean ± SD (*n* = 5; *** *p* < 0.01).

The epidermal tissue of skin is mainly composed of keratin and HaCat cells [35]. Based on the above results, CLD could accelerate the absorption of the hFGF2 protein into skin tissues by changing the structure and content of keratin, but the changes in the morphology of the HaCat cells were unknown. Therefore, atomic force microscopy (AFM) was used to observe the cell height and adhesion force under liquid environments. Compared with PBS treatment, the morphology of the HaCat cells was altered to varying degrees (Figure 5A–D). The peak height of the keratinocytes was 6.15 ± 0.24 μm, 11.5 ± 0.24 μm, 13.25 ± 0.48 μm, and 9.67 ± 0.23 μm under treatment with PBS, CLD, CLD-hFGF2, and hFGF2 freeze-dried powder, respectively (Figure 5E–H). Meanwhile, the adhesion force of the HaCat cells was mainly distributed at 0–3 nN, 0–2.0 nN, 0–2.5 nN, and 0–3.5 nN under treatment with PBS, CLD, CLD-hFGF2, and hFGF2 freeze-dried powder, respectively (Figure 5I–L). Together, these results indicate that CLD-hFGF2 freeze-dried powder could change the height and adhesion force of the HaCat cells. To evaluate whether CLD-hFGF2 freeze-dried powder affects cell morphology by disrupting cell integrity, Triton X-100, a surfactant that can damage the cell membrane, was used as a positive control to evaluate the integrity of the cell membrane.

As shown in Figure 5M–N, the number of damaged cells was significantly increased after treatment with Triton X-100, while the number of damaged cells did not increase with increasing CLD-hFGF2 freeze-dried powder at different times and concentrations. This result showed that CLD-hFGF2 freeze-dried powder changed the cellular morphology it did not damage the integrity of the keratinocytes. Therefore, CLD-hFGF2 freeze-dried powder is able to change the morphology of the HaCat cells and be non-toxic in the process of percutaneous absorption.

### 2.5. CLD-hFGF2 Freeze-Dried Powder Enhances Deep Second-Degree Burn Wound Closure in Rats

**Figure 6:**
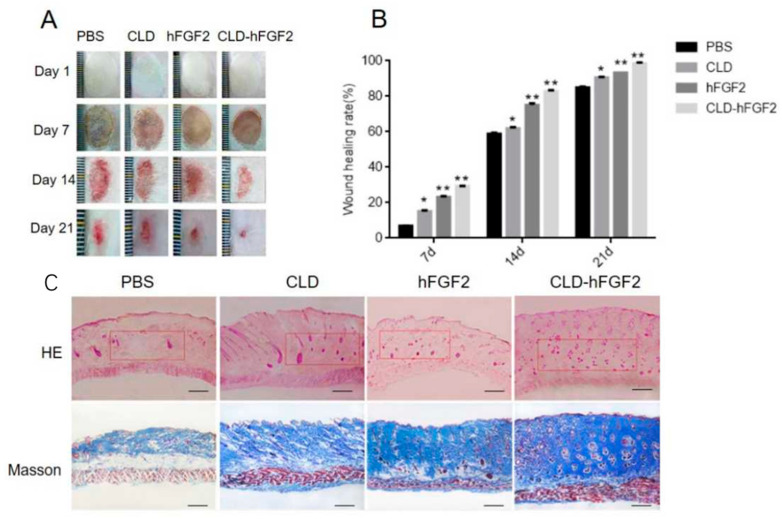
Burn healing assessment for CLD-hFGF2. (**A**) The burn wound under the treatment of PBS, CLD, hFGF2, and CLD-hFGF2 were photographed at days 0, 7, 14, and 21, respectively; (**B**) wound closure rates were counted using Image J at days 0, 7, 14, and 21, respectively; and (**C**) the wound skin tissue was analyzed with HE and Masson staining. The images were shown at 40× magnification. The red boxes represent the effect of the subcutaneous connective tissue and skin appendages (*n* = 15; scale bars = 50 µm; * *p* < 0.05, ** *p* < 0.01, compared with the PBS treatment).

In order to ascertain the biological role of the CLD-hFGF2 freeze-dried powder in second-degree burn healing, a deep second-degree burn on the skin of rats was established, and the process of burn healing was evaluated at 1, 7, 14, and 21 days, respectively. As shown in Figure 6A,B, the CLD-hFGF2 and hFGF2 freeze-dried powder groups demonstrated better wound healing effects than the other groups. Significantly, the area of healing with the presence of CLD-hFGF2 and hFGF2 freeze-dried powder was 98% and 88% at 21 days (*p* < 0.01), respectively; the burn healing was most remarkable with the presence of the CLD-hFGF2 freeze-dried powder. Interestingly, compared with the PBS freeze-dried powder, the CLD freeze-dried powder also had the effect of promoting burn healing (*p* < 0.05). Meanwhile, HE and Masson stains were used to observe the progress of burn healing at 21 days; it could be found that the collagen fibers were more tightly packed and well-integrated with the surrounding region with the presence of the CLD-hFGF2 freeze-dried powder. Therefore, this empathizes that CLD-hFGF2 freeze-dried powder can make the burn healing more perfect through percutaneous absorption.

**Figure 7:**
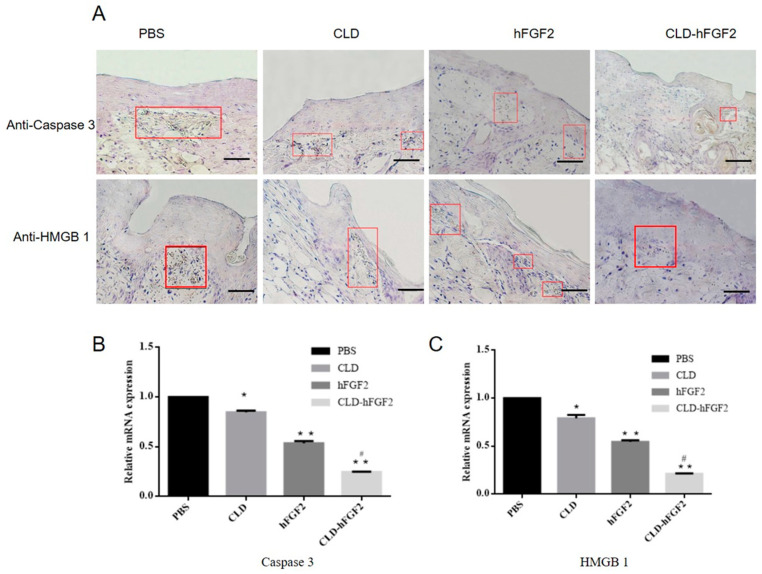
Formation of necrosis and apoptotic cells, and quantitative analysis in the process of burn healing. (**A**) Immunohistochemical staining for Caspase 3 and HNGB1 in the process of burn healing at day 7. The red box shows the corresponding cell changes of total Caspase 3 and HNGB1 during wound healing. (**B**,**C**) Analysis of Caspase 3 and HMGB1 gene expression levels in the process of burn healing at day 7 using qRT-PCR. The images were shown at 20 × 10 magnification (*n* = 3; scale bars = 50 µm; * *p* < 0.05, ** *p* < 0.01, compared with the PBS treatment; ^#^ *p* < 0.05, compared with the hFGF2 treatment).

To verify whether the lipid-soluble drug of the CLD-hFGF2 freeze-dried powder can improve the efficiency of burn healing by inhibiting inflammatory reactions, the expression of the inflammatory factors Caspase 3 and HMGB1 were evaluated and identified during the process of burn wound healing. Immunohistochemistry assays with the burn wound tissues showed that many apoptotic cells surrounded the burn area by the treatment with the control groups at day 21. Interestingly, Caspase 3 and HMGB1 were markedly inhibited with the treatments of CLD and CLD-hFGF2 freeze-dried powder (Figure 7A). In addition, the expression levels of Caspase 3 and HMGB1 genes were significantly decreased in both the hFGF2 and CLD-hFGF2 freeze-dried powder groups (Figure 7B,C). Notably, compared with the hFGF2 group, the expression levels of HMGB1 and Caspase 3 in the CLD-hFGF2 freeze-dried powder group were significantly decreased (*p* < 0.05) (Figure 7B,C). Therefore, CLD can not only generate an easier permeation into the skin for promoting the absorption of hFGF2 in the process of burn wound healing but can also induce a significant inhibitory effect on cell death and reduction in the secretion of inflammatory factors. This may be due to the high content of oleic acid and linoleic acid in the CLD-hFGF2 freeze-dried powder, which can significantly shorten the inflammatory reactions for improving the process of burn wound healing [36].

## 3. Discussion

Plant raw materials have been recently used for numerous applications, including for the synthesis of anti-inflammatory products and cosmetic emulsions [37,38,39,40]. Among the natural materials, fatty vegetable oils (triglycerides) are valuable components of medicinal and decorative cosmetics. As a part of cosmetic compositions, plant fatty acids play the role of emollients, and perform a transport function of the delivery of biologically active substances through the lipid barrier of the skin [41]. The main driving force for the transdermal mode is the hydration gradient, which can produce an osmotic pressure difference. After hydration, permeability was enhanced and the intercellular space of the stratum corneum was dilated. Moreover, plant oil-based emulsion exhibits a higher level of antioxidant activity, and it provides a balance of moisture and fats on the skin for a longer time [42]. Therefore, we speculate that CLD contain a lot of fatty acids to change the skin’s structure and enhance the drug’s permeability into the skin.

As a natural lipid carrier, the lecithin of the CLD membrane can exchange with the skin lipids, increasing the cellular space, making the cuticle structure loose, and increasing the penetration ability of drugs in the skin. Furthermore, an FDA-approved skin penetrant, oleic acid, can effectively improve lipid fluidity into the skin tissues and enter the skin lipids to produce fluidity channels that are easy to penetrate [43,44]. Additionally, CLD-hFGF2 contain lecithin in its monomolecular layer, which can exchange with skin lipids and increase the cellular space, thereby providing a theoretical basis and scientific guidance for the transdermal absorption [15]. Based on the above characteristics, the CLD system can play a positive role during the process of skin administration and is conducive to the passage of the hFGF2 protein through the skin cuticle to further increase the skin penetration ability of drugs.

Although many studies have reported that liposomes can carry foreign proteins and promote their absorption, the correlation between skin tissues has not been reported [45]. In our paper, the melting point of keratin in the epidermis was lower, the peak position of the phase transition peak was changed, and the changes in enthalpy were increased, which directly reflected the change in the keratin and lipid structure after CLD-hFGF2 treatment. This was observed as Camelina lipid droplets contain many unsaturated fatty acids, such as oleic acid and palmitic acid, which can effectively improve the fluidity of the lipids between the cells of the skin tissue to produce easily permeable fluidity pores [46,47]. These data further revealed that CLD-hFGF2 can break through the barrier of the stratum corneum by changing the helical structure of the skin keratin and lipids to achieve percutaneous absorption.

The skin stratum corneum is composed of HaCats, keratins, and lipid layers. The ability of CLDs to break through the barrier of the skin stratum corneum, loosen the tissue of the skin stratum epidermis, and promote the absorption of hFGF2 water-soluble proteins in the skin tissue has been clearly observed from previous studies, but the interaction with skin HaCats is unknown. In this study, the biophysical properties of single cells, including their height and adhesion force, were markedly changed under CLD-hFGF2 treatment, and the CLD system had no destructive effect on HaCat cell integrity. Most liposomes were reported to enter the cells through endocytosis without causing the destruction of the cell membrane integrity [48,49], which is consistent with our findings. These data fully proved that CLD-hFGF2 can affect the shape of HaCats to achieve loose skin tissue.

In this paper, the CLD-hFGF2 treatment significantly promoted wound healing more than the free state of hFGF2. This is due to the fact that the skin wound healing process was accelerated for promoting the absorption of the hFGF2 protein by improving the skin cuticle barrier. Meanwhile, a series of experiments were carried out to explore the absorption mechanism of CLD-hFGF2 in this paper. Under the CLD-hFGF2 treatment, the skin tissue was significantly loosened, and the subcutaneous cumulative release of hFGF2 was twice that of free hFGF2. As a natural lipid carrier, CLD-hFGF2 contains a large amount of unsaturated fatty acids, such as oleic acid and palmitic acid. As an FDA-approved skin penetrant, oleic acid can effectively improve lipid fluidity between the skin tissues and enter skin lipids to produce fluidity channels that are easy to penetrate [50,51]. Therefore, based on the above characteristics, the CLD system can play a positive role during the process of skin administration, and is conducive to the passage of the hFGF2 protein through the skin cuticle to further increase the skin penetration ability of drugs.

In a few words, we focused on clarifying the process regarding the transdermal absorption of CLD-hFGF2 freeze-dried powder in the skin. Regarding the particularity of the CLD component, the absorption effect of the drug in the skin tissue was enhanced in the subcutaneous tissue for accelerating the skin wound healing process by changing the structure of the lipids and keratin, endowing it with unique transport characteristics. This study clarified the enhanced penetration mechanisms of CLD freeze-dried powder, which provides a step for the utilization of the CLD system in transdermal drug delivery both theoretically and practically.

## 4. Materials and Methods

### 4.1. Materials

Transgenic Camelina seeds were preserved by Jilin Agricultural University; the sequence of the hFGF2 gene was obtained from the GenBank (Gene ID: E04331.1) and modified through codon optimization. The hFGF2 was inserted into the pOTB plasmid and the recombinant plasmid was transformed into *Agrobacterium* receptor cells, resulting in the formation of the transgenic Camelina.

### 4.2. Preparation and Characterization of CLD-hFGF2 Freeze-Dried Powder

In order to obtain the CLD-hFGF2, Camelina seeds were ground in a colloid mill with Tris-HCl for 1 min and well crushed. The mixture was then centrifuged and the upper solution underwent emulsion in the Tris-HCl. To obtain the pure CLD-hFGF2, the emulsion was centrifuged at 12,000× *g* and 4 °C for 20 min. The upper emulsion was collected and that it is for CLD-hFGF2.

The CLD-hFGF2 freeze-dried powder was diluted 100 times and then transferred to a copper sheet and dried. The morphology of CLD-hFGF2 freeze-dried powder was analyzed using transmission electron microscopy (TEM: H-600; Hitachi). In addition, the particle size of the CLD-hFGF2 freeze-dried powder was measured with dynamic light scattering analysis (Zetasizer Nano ZS 90, Malvern, UK). The measurements were repeated three times at 25 °C for all samples.

### 4.3. Permeation Assessment of CLD-hFGF2 Freeze-Dried Powder In Vitro

BALB/c mice were fed for one week, and their dorsal hairs were removed with depilate cream. They were sacrificed, and the dorsal skin without subcutaneous fatty tissue was obtained. CLD-hFGF2 (30 μg), hFGF2 (30 μg), and PBS (pH = 7.0) were added to the Franz (Franz, Shanghai Yuyan Scientific Instrument Co., Ltd., shanghai), and the effective contact area was 1.55 cm^2^. In the in vitro permeation study, the CLD-hFGF2 freeze-dried powder (30 μg), hFGF2 freeze-dried powder (30 μg), and PBS groups were used, and the cumulative release of hFGF2 in the subcutaneous tissue was also evaluated. ELISA was used to detect the reception pool under predetermined intervals (60, 90, 120, and 150 min, respectively). The calculation formula is as follows:

*M_n_* = [*Cn* × *V* + ∑(*C_i_V_i_*)]/*A*

where A is the effective area of skin contact, *V* is the volume of normal saline, *V_i_* is the volume of samples taken at each time point, *C_i_* is the ith measured drug concentration, I = 60, 90, 120, and 150 min, *C_n_* is the Nth measured drug concentration, and *M_n_* denotes the cumulative amount of skin absorption and permeability of the hFGF2 protein at different time points.

### 4.4. Histopathological Analysis of the Skin Cuticle Structure 

BALB/c mice were purchased from YiSi Biotechnology Limited Company (Changchun, China). They were randomly divided into four groups (*n* = 12 per group). Chloral hydrate (0.1 mL/10 g) was injected into the abdominal cavity of the mice. The dorsal hairs of the BALB/c mice were shaved using the depilator, and then a small amount of depilation cream was applied to remove the dorsal hairs of the mice. Drug treatment: samples from the hFGF2 freeze-dried powder (30 μg and 200 μL) group, CLD-hFGF2 freeze-dried powder (30 μg and 200 μL) group, and PBS freeze-dried powder group (200 μL) were smeared onto the back skin of the mice, and the absorption effect of the CLD-hFGF2 freeze-dried powder in the tissues was observed at 60 min, 90 min, 120 min, and 150 min, respectively. The skin tissues of the BALB/c mice were collected and soaked in 4% paraformaldehyde. The skin was cut to a 0.5 μm thickness for H&E staining and was observed using a fluorescence inverted microscope (IX51, OLYMPUS, Tokyo, Japan).

### 4.5. Analysis of Skin Cuticle Thermotropic Properties with Differential Scanning Calorimetry (DSC)

The dorsal hairs of the BALB/c mice were removed; drug treatment consisted of the following: samples from the hFGF2 freeze-dried powder (30 μg and 200 μL) group, CLD-hFGF2 freeze-dried powder (30 μg and 200 μL) group, and PBS freeze-dried powder group (200 μL) were smeared onto the back skin of the mice. After treatment for 60 min, tissue residues were removed with clean water and the skin tissues of the mice were then stripped. To remove moisture, the skin tissue was pre frozen in a −80 °C refrigerator, and the pre-frozen skin tissue was transferred into a freeze-drying machine for drying treatment after 12 h. The freeze-drying parameters of the freeze-drying machine: the temperature of the cold trap is −45 °C, the temperature above the cold trap is about 0 °C, the vacuum degree is 10 Pa, and the freeze-drying time is set to 30 h. After that, the dry skin tissue was used for thermodynamic analysis with DSC (Q2000, Waters, American). The skin tissues of the mice were peeled off for thermodynamic analysis under the conditions of a 20–150 °C temperature and a temperature rise rate of 10 °C.min^−1^.

### 4.6. Analysis of the Skin Cuticle Components Using Fourier Transform Infrared Spectroscopy (FTIR)

The dorsal hairs of the BALB/c mice were removed; drug treatment consisted of the following: samples from the hFGF2 freeze-dried powder (30 μg and 200 μL) group, CLD-hFGF2 freeze-dried powder (30 μg and 200 μL) group, and PBS freeze-dried powder group (200 μL) were smeared onto the back skin of the mice. After treatment for 60 min, the residual samples on the skin were washed using clean water. The skin tissue was removed and ground into powder under liquid nitrogen conditions. Then, a Fourier transform infrared spectrometer (Nicolet 6700 FT-IR, Thermo Fisher Scientific, USA) was used to scan the infrared spectrum of the cuticle with a photovoltaic MCT detector at 37 °C. The scanning wave number was 600~4000 cm^−1^, the resolution was 2 cm^−1^, and the scanning number was 20.

### 4.7. Atomic Force Microscope Assay

HaCat cells were seeded into 6-well plates (4000 cells per well) and cultured overnight in 10% PBS. Fresh medium was added to replace the old medium, and PBS freeze-dried powder, CLD freeze-dried powder, hFGF2 freeze-dried powder (100 ng/mL), and CLD-hFGF2 freeze-dried powder (100 ng/mL) were added to the culture for 24 h. The height and adhesion force of the cells were observed using atomic force microscopy (AFM). AFM measurements, using an Agilent Technologies 5500 Scanning Probe Microscope (SPM, Agilent Technologies Company, Palo Alto, CA, USA) and JPK (NanoWizard^®^3, JPK instruments, Berlin, Germany), were performed.

### 4.8. Deep Second-Degree Burn Wound Model and Treatment

Wistar rats (n = 60, 200–220 g) were purchased from Yisi Biotechnology Limited Company (Changchun, China), raised to adapt to the environment (23 ± 2 °C, humidity: 40–60%) for a week, and were provided adequate commercial feed and water. Sixty male adult Wistar rats were randomly divided into four groups, and sodium pentobarbital at a 0.06 mg/g body weight was intraperitoneally injected for anesthesia. At the same time, the hairs with the backside were shaved and used to make the burn model. A brass hot block (with a diameter of 2 cm and weight of 100 g) was used to cause contact burns on the back of the rat. The hot block was preheated in temperature-controlled boiling water set at 100 °C. It was deemed to be convenient to prepare burn wounds of the same degree by keeping the brass hot block on the skin for 5 s. Then, these rats were divided into four groups: the blank control group (PBS freeze-dried powder), negative group (CLD freeze-dried powder: 8.75 mg/mL total protein), positive group (hFGF2 freeze-dried powder: 0.25 mg/mL total protein), and treatment group (CLD-hFGF2 freeze-dried powder: the total protein of 10 mg/mL with contain the hFGF2 was 0.25 mg), and every treatment was added as a 400 uL volume on the burn area every day.

To evaluate the healing of the skin tissue after drug treatment, all the rats were sacrificed with intraperitoneal injections of 0.1 mg/g body weight pentobarbital sodium at 7, 14, and 21 days after injury. Images of the wounds were obtained at 7, 14, and 21 days after healing and were analyzed using Image J software. Wound healing rate = (initial wound area-final wound area)/initial wound area × 100%. Animal care and experimental protocols were approved by the Department of Laboratory Animal Resources, Jilin Agricultural University Ethics Committee (No. 2019.06.20001).

### 4.9. Quantitative Real-Time PCR

Burn skin tissue RNA was extracted using Trizol reagent (Thermo Fisher science company of the United States). Gene-specific primers for HMGB1, Caspase 3, and GAPDH were designed. The forward primer of GAPDH: TCCCTCAAGATTGTCAGCAA, and the reverse primer of GAPDH: AGATCCACAACGGATACATT. The forward primer of HMGB1: ATGGGCAAAGGAGATCCTA, and the reverse primer of HMGB1: ATTCATCATCATCATCTTCT. The forward primer of Caspase 3: ATGGAGAACAATAAAACCT, and the reverse primer of Caspase 3: CTAGTGATAAAAGTAGAGTTC. Quantitative experiments were set for three times, and three parallel experiments were set for each experiment.

### 4.10. Statistical Analysis

All statistical data were analyzed in the format of mean ± standard deviation. Student’s t-tests were used to determine the significant differences between the groups, with * *p* < 0.05 and ** *p* < 0.01 considered as statistically significant and highly significant, respectively.

## Data Availability

Data are contained within the article.

## References

[1] Homaeigohar S., Boccaccini A.R. (2020). Antibacterial Biohybrid Nanofibers for Wound Dressings. Acta Biomater..

[2] Kaur R., Sharma N., Tikoo K., Sinha V.R. (2020). Development of mirtazapine loaded solid lipid nanoparticles for topical delivery: Optimization, characterization and cytotoxicity evaluation. Int. J. Pharm..

[3] Watkinson A.C., Kearney M.C., Quinn H.L., Courtenay A.J., Donnelly R.F. (2016). Future of the transdermal drug delivery market have we barely touched the surface. Expert Opin. Drug Deliv..

[4] Karande P., Jain A., Mitragotri S. (2006). Insights into synergistic interactions in binary mixtures of chemical permeation enhancers for transdermal drug delivery. J. Control Release.

[5] Milewski M., Yerramreddy T.R., Ghosh P., Crooks P.A., Stinchcomb A. (2010). In vitro permeation of a pegylated naltrexone prodrug across microneedle-treated skin. J. Control Release.

[6] Wu X.M., Todo H., Sugibayashi K. (2007). Enhancement of skin permeation of high molecular compounds by a combination of microneedle pretreatment and iontophoresis. J. Control Release.

[7] Polat B.E., Hart D., Langer R., Blankschtein D. (2011). Ultrasound-mediated transdermal drug delivery: Mechanisms, scope, and emerging trends. J. Control Release.

[8] Sammeta S.M., Vaka S.R., Murthy S. (2010). Transcutaneous electroporation mediated delivery of doxepin-HPCD complex: A sustained release approach for treatment of postherpetic neuralgia. J. Control Release.

[9] Cevc G., Vierl U. (2010). Nanotechnology and the transdermal route: A state of the art review and critical appraisal. J. Control Release.

[10] Gao H., Wang F., Hu X., Li Y., Zhang Y., Carther K.F.I., Wang B., Min F., Wang X., Wu H. (2021). Cameline Lipid Droplets as skin delivery system promotes wound repair by enhancing the absorption of hFGF2. Int. J. Pharm..

[11] Qiang W., Zhou T., Lan X., Zhang X., Guo Y., Noman M., Du L., Zheng J., Li W., Li H. (2018). A new nanoscale transdermal drug delivery system: Oil body-linked oleosin-hEGF improves skin regeneration to accelerate wound healing. J. Nanobiotechnology.

[12] Horn P.J., James C.N., Gidda S.K., Kilaru A., Dyer J.M., Mullen R.T., Ohlrogge J.B., Chapman K.D. (2013). Identification of a new class of lipid droplet-associated proteins in plants. Plant Physiol..

[13] Li X., Mupondwa E. (2014). Life cycle assessment of camelina oil derived biodiesel and jet fuel in the Canadian Prairies. Sci. Total Environ..

[14] Yang J., Guan L., Guo Y., Du L., Wang F., Wang Y., Zhen L., Wang Q., Zou D., Chen W. (2015). Expression of biologically recombinant human acidic fibroblast growth factor in Arabidopsis thaliana seeds via oleosin fusion technology. Gene.

[15] Shah P.P., Desai P.R., Singh M. (2012). Effect of oleic acid modified polymeric bilayered nanoparticles on percutaneous delivery of spantide II and ketoprofen. J. Control Release.

[16] Tanojo H., Bos-van Geest A., Bouwstra J.A., Junginger H.E., Boodé H.E. (1997). In vitro human skin barrier perturbation by oleic acid: Thermal analysis and freeze fracture electron microscopy studies. Thermochim. Acta.

[17] Mondor M., Hernández-Lvarez A.J. (2021). Camelina Sativa Composition, Attributes, and Applications: A Review. Eur. J. Lipid Sci. Technol..

[18] Zanetti F., Alberghini B., Jeromela A.M., Grahovac N., Rajković D., Kiprovski B., Monti A. (2021). Camelina, an ancient oilseed crop actively contributing to the rural renaissance in Europe. A review. Agron. Sustain. Dev..

[19] Kim N., Li Y., Sun X.S. (2015). Epoxidation of Camelina sativa oil and peel adhesion properties. Ind. Crops Prod..

[20] Waraich E.A., Ahmed Z., Ahmad R., Ashraf M., Naeem M.S., Rengel Z. (2013). Camelina sativa, a climate proof crop, has high nutritive value and multiple-uses: A review. Aust. J. Crop Sci..

[21] Righini D., Zanetti F., Martinez-Force E., Mandrioli M., Toschi T.G., Monti A. (2019). Shifting sowing of camelina from spring to autumn enhances the oil quality for bio-based applications in response to temperature and seed carbon stock. Ind. Crops Prod..

[22] Righini D., Zanetti F., Monti A. (2016). The bio-based economy can serve as the springboard for camelina and crambe to quit the limbo. OCL.

[23] Zhao Y.Z., Tian X.Q., Zhang M., Cai L., Ru A., Shen X.-T., Jiang X., Jin R.-R., Zheng L., Hawkins K. (2014). Functional and pathological improvements of the hearts in diabetes model by the combined therapy of bFGF-loaded nanoparticles with ultrasound-targeted microbubble destruction. J. Control Release.

[24] Alemdaroğlu C., Degim Z., Celebi N., Şengezer M., Alömeroglu M., Nacar A. (2008). Investigation of epidermal growth factor containing liposome formulation effects on burn wound healing. J. Biomed. Mater Res. A.

[25] Bartolini B., Caravà E., Caon I., Parnigoni A. (2020). Heparan Sulfate in the Tumor Micro-environment. Adv. Exp. Med. Biol..

[26] Van Rooijen G.J.H., Moloney M.M. (1995). Plant seed oil-bodies as carriers for foreign proteins. Bio-Technology.

[27] Deckers H., Moloney M.M., Baum A. (1999). The case for recombinant production of pharmaceutical proteins in plants. Annu. Rep. Med. Chem..

[28] Markley N., Nykiforuk C., Boothe J., Moloney M. (2006). Producing proteins using transgenic oilbody oleosin technology. BioPharm. Int..

[29] Boothe J., Nykiforuk C., Shen Y., Zaplachinski S., Szarka S., Kuhlman P., Murray E., Morck D., Moloney M.M. (2010). Seed-based expression systems for plant molecular farming. Plant Biotechnol. J..

[30] Yang Y., Xia T., Chen F., Wei W., Liu C., He S., Li X. (2012). Electrospun Fibers with Plasmid hFGF2 Polyplex Loadings Promote Skin Wound Healing in Diabetic Rats. Mol. Pharm..

[31] Cotte M., Dumas P., Besnard M., Tchoreloff P., Walter P. (2004). Synchrotron FT-IR microscopic study of chemical enhancers in transdermal drug delivery: Example of fatty acids. J. Control Release.

[32] Kumar P., Singh S.K., Handa V., Kathuria H. (2018). Oleic Acid Nanovesicles of Minoxidil for Enhanced Follicular Delivery. Medicines.

[33] Gu Y., Yang M., Tang X., Wang T., Yang D., Zhai G., Liu J. (2018). Lipid nanoparticles loading triptolide for transdermal delivery: Mechanisms of penetration enhancement and transport properties. J. Nanobiotechnology.

[34] Shakeel F., Baboota S., Ahuja A., Ali J., Shafiq S. (2008). Skin permeation mechanism and bioavailability enhancement of celecoxib from transdermally applied nanoemulsion. J. Nanobiotechnology.

[35] Poljšak N., Kreft S., Kočevar Glavač N. (2020). Vegetable butters and oils in skin wound healing: Scientific evidence for new opportunities in dermatology. Phytother. Res..

[36] Yang M., Gu Y., Yang D., Tang X., Liu J. (2017). Development of triptolide-nanoemulsion gels for percutaneous administration: Physicochemical, transport, pharmacokinetic and pharmacodynamic characteristics. J. Nanobiotechnology.

[37] Maione F., Russo R., Khan H., Mascolo N. (2016). Medicinal plants with anti-inflammatory activities. Nat. Prod. Res..

[38] Chu C., Nyam K. (2020). Kenaf (*Hibiscus cannabinus* L.) seed oil: Application as cosmetic product ingredients. Ind. Crops Prod..

[39] Saribekova D., Kunik O., Harhaun R., Saleba L., Cavallaro G. (2021). The use of silicones as extractants of biologically active substances from vegetable raw materials. Appl. Sci..

[40] Mahbub K., Octaviani I., Astuti I., Sisunandar S., Dhiani B. (2022). Oil from *kopyor* coconut (*Cocos nucifera* var. *Kopyor*) for cosmetic application. Ind. Crops Prod..

[41] Sala M., Diab R., Elaissari A., Fessi H. (2018). Lipid nanocarriers as skin drug delivery systems: Properties, mechanisms of skin interactions and medical applications. Int. J. Pharm..

[42] Kunik O., Saribekova D., Lazzara G., Cavallaro G. (2022). Emulsions based on fatty acid from vegetable oils for cosmetics. Ind. Crops Prod..

[43] Zhai Y., Zhao L., Wang Z., Zhai G. (2014). RETRACTED ARTICLE: Preparation and characterization of novel lipid nanocapsules of ropivacaine for transdermal delivery. Drug Deliv..

[44] Staiger K., Staiger H., Weigert C., Haas C., Häring H.U., Kellerer M. (2006). Saturated, but not unsaturated, fatty acids induce apoptosis of human coronary artery endothelial cells via nuclear factor-kappaB activation. Diabetes.

[45] Zhai Y., Yang X., Zhao L., Wang Z., Zhai G. (2014). Lipid nanocapsules for transdermal delivery of ropivacaine: In vitro and in vivo evaluation. Int. J. Pharm..

[46] Kumar M., Sharma G., Singla D., Singh S., Sahwney S., Chauhan A.S., Singh G., Kaur I.P. (2014). Development of a validated UPLC method for simultaneous estimation of both free and entrapped (in solid lipid nanoparticles) all-trans retinoic acid and cholecalciferol (vitamin D3) and its pharmacokinetic applicability in rats. J. Pharm. Biomed. Anal..

[47] Puglia C., Offerta A., Tirendi G.G., Tarico M.S., Curreri S., Bonina F., Perrotta R.E. (2016). Design of solid lipid nanoparticles for caffeine topical administration. Drug Deliv..

[48] Li H., Wen X.S., Di W. (2012). In vitro and in vivo evaluation of Triptolide-loaded pluronic P105 polymeric micelles. Arzneimittelforschung.

[49] Jia Y., Liu J., Xu J. (2018). Influence of grapefruit juice on pharmacokinetics of triptolide in rat’s grapefruit juice on the effects of triptolide. Xenobiotic.

[50] Anderson J.V., Wittenberg A., Li H., Berti M. (2019). High throughput phenotyping of *Camelina sativa* seeds for crude protein, total oil, and fatty acids profile by near infrared spectroscopy. Ind. Crops Prod..

[51] Fang C.L., Al-Suwayeh S.A., Fang J.Y. (2013). Nanostructured lipid carriers (NLCs) for drug delivery and targeting. Recent Pat. Nanotechnol..

